# A Case Series of Bee Sting Keratopathy With Different Outcomes in Malaysia

**DOI:** 10.7759/cureus.1035

**Published:** 2017-02-17

**Authors:** Wen-Jeat Ang, Siti-Zakiah MD Kadir, Abdul-Jalil Fadzillah, Embong Zunaina

**Affiliations:** 1 Department of Ophthalmology, Universiti Sains Malaysia; 2 Department of Ophthalmology, Melaka General Hospital

**Keywords:** cornea bee sting, toxic keratopathy, bee, wasp, bee sting keratopathy

## Abstract

We report three patients with corneal bee sting at our tertiary care center in a three-year period starting from 2014 to 2016. All patients sustained a bee sting injury to the cornea. All patients received early preoperative topical antibiotics, topical cycloplegic and intensive topical steroids. However, the timing of the initial presentation, the duration, and the location of the retained stinger differed in each case leading to different postsurgical outcomes.

## Introduction

Corneal bee sting injuries are relatively infrequent and is often associated with a myriad of visually debilitating ocular complications. The effects of bee sting corneal injury is often a challenging one to tackle, causing debilitating effects to vision despite sufficient medical and surgical intervention. Here, we present a case series of cornea bee sting with varied visual outcomes. Informed consent was obtained from all the patients for this study.

## Case presentation

### Case 1

A 44-year-old female presented with severe pain, tearing, redness, and reduced vision 12 hours after being stung by a bee over the right eye. The best corrected visual acuity (BCVA) in the right eye was 6/60 and in the left eye was 6/6. An ocular examination revealed a retained barbed stinger that was embedded at the center of the cornea, which had a large epithelial defect that might have occured due to the patient rubbing her eye after the cornea bee sting. The cornea was oedematous with generalised Descemet striae. There was moderate anterior chamber inflammation with 2 mm level of hypopyon. The iris appeared to be generally atrophied with no cataract. The posterior segment could not be visualized due to severe corneal oedema. However, a B-scan ultrasound of the globe was normal. She received intensive topical dexamethasone 0.1% quarter hourly along with topical moxifloxacin 0.5% hourly. Topical homatropine two percent three times a day was also added. This was followed by the removal of the bee stinger three hours later. The bee stinger was removed after performing a small corneal incision from the base to the apex over the entire length of the stinger plane. Post operation, topical steroids and topical antibiotic were continued and were tapered down gradually over a two-month period. At the sixth-month follow-up, her visual acuity was only counting fingers at 1 ft due to a dense leucomatous central cornea scar and the development of an anterior subcapsular cataract.

### Case 2

A 36-year-old man presented with a left corneal bee sting six hours after an alleged foreign body entry while gardening. He complained of severe eye pain, redness, tearing, and blurring of vision over the affected eye. On examination, the visual acuity in his left eye was 6/60, improving to 6/24 with pinhole. A barbed bee stinger with surrounding deep corneal stromal infiltration was seen paracentrally. There were generalised cornea Descemet's membrane striae and a mid-dilated pupil. The posterior segment could not be visualized due to severe corneal oedema. However, a B-scan ultrasound of the globe showed no abnormalities. He was treated with intensive topical dexamethasone 0.1% quarter hourly and topical moxifloxacin 0.5% hourly. About two hours post presentation, the bee stinger was removed under local anaesthesia after performing a small corneal incision from the base to the apex over the entire length of the stinger plane. Post removal of the bee stinger, topical steroids and topical antibiotic were continued and were tapered down gradually over a six-week period. A subsequent follow-up at six months showed a visual acuity of 6/36 due to a dense paracentral cornea scar.

### Case 3

**Figure 1:**
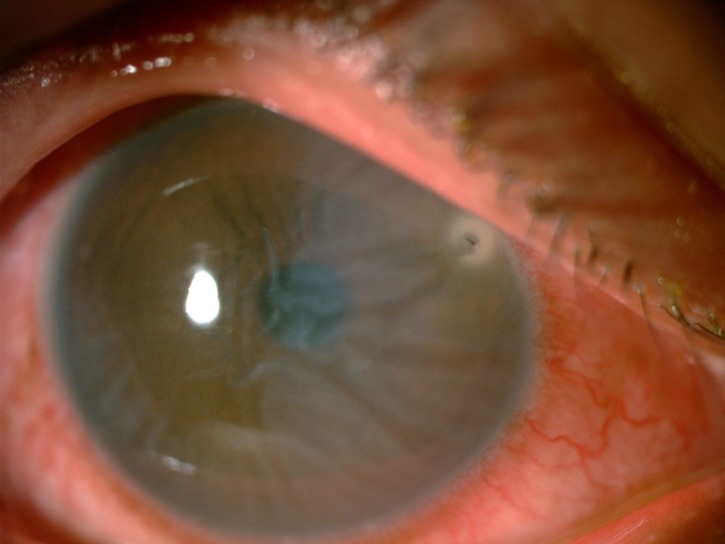
Bee stinger at the two o'clock region, 1 mm from the limbus with Descemet striae over the nasal half of the cornea.

**Figure 2:**
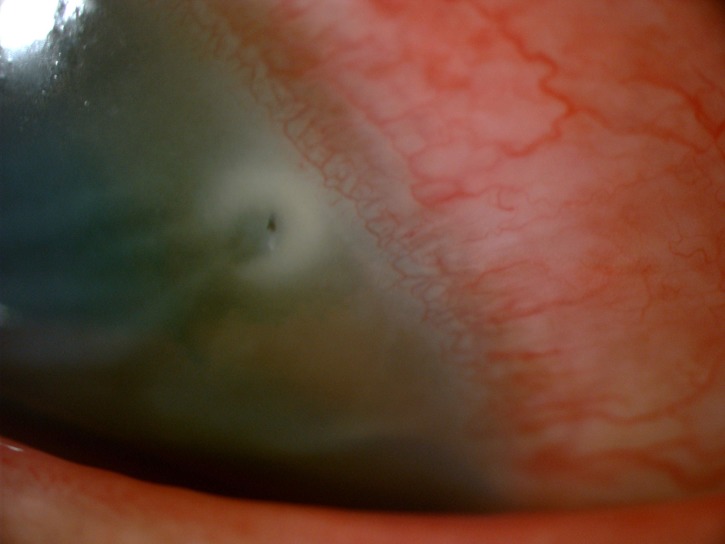
Surrounding corneal stromal infiltrates.

**Figure 3:**
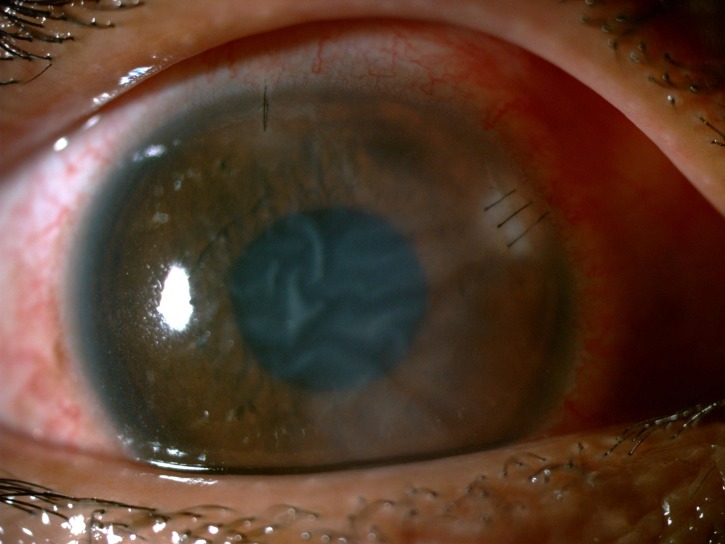
Image showing marked improvement of corneal infiltrates and oedema of the right eye at three days post removal of the bee stinger.

**Figure 4:**
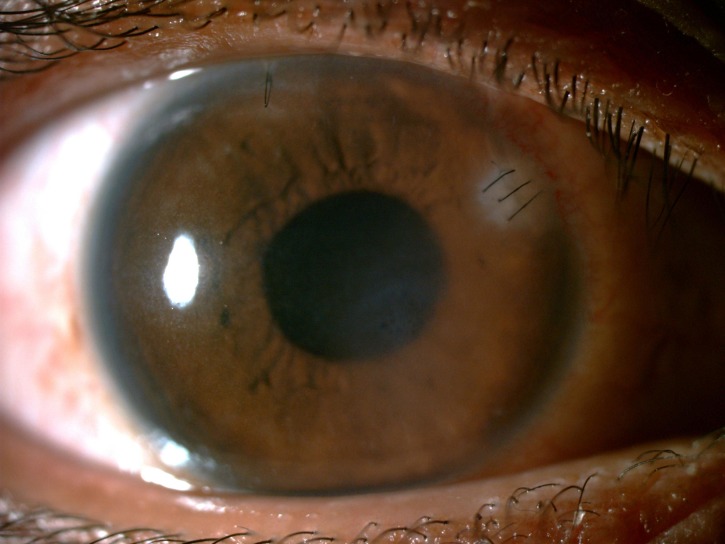
Clear cornea with scar formation at the embedded bee stinger area at the one-month follow-up.

A 26-year-old man complained of a foreign body entry into his right eye while he was driving. He presented four hours later with excruciating pain, redness, photophobia, tearing, and blurring of vision over the affected eye. His vision on presentation was hand movement over the right eye and 6/6 over the left eye. An examination of his right eye revealed generalised conjunctival congestion. There was a deeply embedded bee stinger at the two o'clock region 1 mm from the limbus (Figure 1). There were surrounding corneal infiltrates (Figure 2) and corneal oedema with Descemet striae involving the nasal half of the cornea. The anterior chamber was deep with the presence of flare. The pupils were reactive with clear lens. A funduscopic examination revealed clear vitreous with normal optic disc and retina. The patient was started on intensive topical dexamethasone 0.1% quarter hourly and topical moxifloxacin 0.5% hourly. The bee stinger was removed after performing a small corneal incision from the base to the apex over the entire length of the stinger plane four hours after the incident. Three days later there was resolution of cornea infiltrates and significant improvement of corneal clarity (Figure 3). Topical steroids and topical antibiotic were continued and were tapered down gradually over a two-month period (Figure 4). A follow-up of the patient six months later revealed a favourable 6/6 visual acuity with clear cornea and scar formation at the embedded bee stinger area.

## Discussion

The ocular complications following bee sting injuries are attributed to either mechanical, immunology, or toxic venom effect or as a combination of all three mechanisms. Ocular bee sting injuries involving the cornea commonly present with corneal oedema, infiltrates, striate keratopathy, and bullous keratitis [1-2]. Isolated case reports have also described anterior uveitis, hyphema, iris atrophy, cataract, optic neuritis, and ophthalmoplegia [3-7]. In all of our patients, the cornea bee stinger was embedded deep stromally, and a characteristic striate keratitis which is a pathognomonic finding in bee sting keratopathy was present. These complications are attributed to the biologic amines, low molecular weight peptide, major protein subunits and enzymes in the bee venom [1].

Due to the scarcity of the injury there are no established evidence-based guidelines for its management. It is generally accepted that the removal of the bee stinger is required in cases that are complicated with corneal infiltration and oedema, more so if the stinger is deeply seated [8-9]. However, it still remains controversial as to whether the bee stinger should be removed, and if so, when and how should it be done. An attempt to remove often results in more inflammation due to additional venom discharge during manipulation. A number of reports also found that the stinger remained inert and could safely be left behind if the patient is asymptomatic [5,10]. To the contrary, the amount of toxin released correlates with the length of the time that the stinger remains within the tissue. This spurred several suggestions that the embedded stinger should be removed as soon as possible [1]. In all of our patients, we commenced early intensive topical corticosteroids to combat the venom-induced inflammation and a cycloplegic to reduce the ciliary spasm and stabilize the blood-aqueous barrier. Also, topical broad spectrum antibiotics are used prophylactically to prevent an infection. Based on the literature thus far, no authors have advocated intracameral or intravitreal steroids in the management of an ocular bee sting injury. The removal of the bee stinger can be done under local anaesthesia, if patient cooperation is expected during surgery. We made a corneal incision from the base to the apex over the entire length of the stinger plane as we found that this allows an easier route of removal. This could be due to the barbed anatomy of the bee stinger. Lin, et al. showed that removal of the bee stinger manually with forceps alone can cause retention of stinger fragments within the cornea [4]. Postoperatively, topical steroids and broad spectrum antibiotics were continued. The variable outcome six months after could be attributed to several factors. It appears that the location of the stinger does affect the final visual outcome evidenced by good visual outcomes in Case 2 and 3, which were paracentral and peripheral, respectively. The final poor visual acuity in Case 1 can be attributed to both the centrally located stinger and secondary cataract. The differences in the time of presentation are also contributary, as the amounts of venom released could be more in the first two patients. This venom contains substances that are toxic to the corneal endothelial cells [9]. The endothelial cell density decreased rapidly despite intensive topical corticosteroid, leading to cornea decompensation and scarring later on.

## Conclusions

Though cornea bee sting injuries are rare occurrences, they are often associated with potential severe visual impairment. An immediate and prompt surgical intervention to remove the bee stinger and concurrent intensive steroid therapy can curtail the toxic effects of a bee sting and hence improve visual outcomes.
